# Disability doesn’t discriminate: health inequities at the intersection of race and disability

**DOI:** 10.3389/fresc.2023.1075775

**Published:** 2023-07-06

**Authors:** Brooke Dorsey Holliman, Michelle Stransky, Nathalie Dieujuste, Megan Morris

**Affiliations:** ^1^ Department of Family Medicine, University of Colorado School of Medicine, Aurora, CO, United States; ^2^ Adult and Child Center for Health Outcomes Research and Delivery Science (ACCORDS), University of Colorado, Aurora, CO, United States; ^3^ Department of Medicine, Boston Medical Center, Boston, MA, United States; ^4^ Department of Psychology, University of Denver, Denver, CO, United States; ^5^ Department of General Internal Medicine, University of Colorado School of Medicine, Aurora, CO, United States

**Keywords:** disability, discrimination, BIPOC, disparities (health, racial), survey data

## Abstract

**Objectives:**

Given the prevalence of discrimination experienced by racial and ethnic minorities living with disabilities, it is likely that racism experienced by Black, Indigenous, and people of color (BIPOC) is compounded by the ableism experienced by people with disabilities, widening disparities in health and healthcare outcomes. To address this, we described unmet healthcare needs of a sample of Black, non-Hispanic, and Hispanic adults with and without disabilities. The following research question was examined exploratively: Are Black and Hispanic adults with disabilities at increased risk of unmet healthcare needs compared to Black and Hispanic adults without disabilities according to the 2018 National Health Interview Survey?

**Methods:**

Survey data was examined from the 2018 National Health Interview Survey (NHIS), a nationally representative survey of community-dwelling adults in the United States.

**Results:**

Black and non-Hispanic adults most commonly reported mobility only disabilities. People with disabilities were significantly more likely to delay or forego care than their peers without disabilities within each racial/ethnic group. Among non-Hispanic Black and Hispanic adults, nearly 30% of people with disabilities forewent services due to cost compared to persons without disabilities.

**Conclusions:**

Black and Hispanic adults with disabilities experience greater disparities in access to healthcare than Black and Hispanic adults without disabilities. Therefore, health disparities experienced by racial and ethnic minorities living with disabilities is likely influenced by the dual systemic factors of racism and ableism.

## Introduction

Recent estimates indicate that 26% of US adults experience disability (1), with higher rates of disabilities in Black, Indigenous, and people of color (BIPOC) communities. For example, compared to 26.6% of white persons with disabilities (PwD) ages 45–64, 35.5% of Black and Hispanic adults in that same age group are living with a disability in the US (2). Moreover, research shows that both BIPOC and disability communities experience disparities in the receipt of equitable care (3–5). For example, each report unmet needs for healthcare at disproportionately higher levels than their white, non-Hispanic and non-disabled counterparts, respectively (2, 6, 7). Both groups face insurance, cost, and provider-patient communication barriers to high-quality care and problems receiving culturally appropriate care.

Despite the overlap in both prevalence and similar inequities experienced by both groups, little research has examined the health and health care outcomes at the intersection of BIPOC and PwD. Given the prevalence of discrimination, it is likely that racism experienced by BIPOC is compounded by the ableism experienced by PwD, potentially amplifying disparities in health and healthcare outcomes (8–10). Studies have demonstrated in specific disability populations, namely individuals with intellectual and developmental disabilities, who belong to racial and ethnic marginalized communities have worse health and health care outcomes as compared to white individuals with intellectual and developmental disabilities (11–15). Additionally, several studies demonstrate disparities in health care utilization for women who live at the intersection of disability and race/ethnicity (16, 17). However, a dearth of evidence on the health care outcomes of the population of persons with disabilities who belong to ethnic and racial minority communities exists. To address this, we described unmet healthcare needs of a sample of Black and Hispanic adults with and without disabilities. The following research question was examined exploratively: Are Black and Hispanic adults with disabilities at increased risk of unmet healthcare needs compared to Black and Hispanic adults without disabilities according to the 2018 National Health Interview Survey?

## Methods

We examined data from the 2018 National Health Interview Survey (NHIS), a nationally representative survey of community-dwelling adults in the US (18). The NHIS is a longstanding in-person federal survey of health and healthcare; it is publicly available from the National Center for Health Statistics and is, therefore, exempt from review. All persons in 30,000 households were interviewed; a single adult was randomly selected for more extensive questions on the Sample Adult file (*n* = 25,417). The response rate on this file was 83.9% (18).

### Sample

We focus our analysis on Black, non-Hispanic adults and Hispanic adults with and without disabilities, due to the small racial/ethnic sample sizes for other BIPOC. Our retrospective cohort study includes 2 analytic samples (≥18 years old): Black, non-Hispanic adults (*n* = 2,822) and Hispanic adults of any race (*n* = 3,069).

Respondents were categorized as having a disability if they reported a lot of difficulty in any of the following areas: seeing with glasses; hearing with a hearing aid; walking or climbing steps; communicating in their “usual language”; “remembering or concentrating”; or self-care activities. Combining disability types into a single catch-all category is not ideal, because of how types of disability interact differently with the environment (19). However, the NHIS first asked these questions of all sample adults in 2018 and the sample sizes of each disability type were too small to be examined reliably. When possible, we describe some preliminary differences by disability type.

### Measures

Outcome measures included self-reported usual source of care (USC) for sick and routine care. Respondents reported 4 separate measures of unmet need: (1 and 2) delayed/forewent care due to cost, (3) delayed care due to availability (e.g., could not get through on the phone, wait was too long), and (4) forewent services (e.g., prescription medication, mental healthcare) due to cost.

### Analysis

Pearson's *χ*^2^ and multivariate logistic regression were used to examine outcomes by disability within each race/ethnicity group, controlling for age, sex, marital status, employment, education, imputed poverty status, health insurance, and number of chronic conditions. We report predicted proportions and odds ratios (95% confidence intervals) from regression. Analyses were conducted in Stata 15, accounting for NHIS weights and complex sampling design. We used *p* < 0.017 to account for the examination of multiple outcomes within each racial/ethnic group (20).

## Results

Sociodemographic characteristics of our sample are contained in Table 1. Fifty percent of adults were ages 35–64 and female; most had private health insurance, graduated high school or earned a GED, and were employed. Approximately 20% of adults reported living in poverty. Most had no chronic conditions.

**Table 1:** Characteristics of black, non-Hispanic and Hispanic adults in the sample.

	Black, non-Hispanic		Hispanic	
	%	(*n*)	%	(*n*)
Total		(2,822)		(3,069)
Age				
18–34	35.1	(702)	39.4	(1,033)
35–64	49.3	(1,396)	49.5	(1,528)
>64	15.6	(724)	11.0	(508)
Female	54.1	(1,672)	50.6	(1,757)
Married	32.9	(728)	49.1	(1,354)
Employed	61.9	(1,579)	66.5	(1,954)
Education				
Less than high school	13.2	(446)	27.7	(881)
High school graduate/GED	28.7	(785)	27.1	(797)
Some college	22.4	(605)	17.4	(482)
College degree	35.7	(986)	27.7	(909)
Poverty status^a^				
<100% FPL^b^	20.6	(581)	18.0	(542)
100%–199% FPL	23.5	(537)	28.6	(708)
200%–399% FPL	28.7	(658)	30.5	(743)
≥400% FPL	27.2	(558)	22.9	(586)
Health insurance				
None	11.7	(300)	23.6	(677)
Public only	28.1	(922)	24.4	(844)
Any private	60.2	(1,600)	52.0	(1,548)
Chronic conditions				
None	43.6	(1,031)	58.4	(1,659)
1	28.2	(745)	23.1	(713)
≥2	28.2	(1,046)	18.6	(697)

^a^ Poverty Status was imputed by the National Center for Health Statistics. Cell sizes reflect unimputed frequencies.

^b^ *FPL*, Federal Poverty Level.

Approximately 11.5% (*n* = 402) of Black, non-Hispanic adults reported a disability; the most common disabilities were mobility only disabilities [4.7% (*n* = 197)] and multiple disabilities, including mobility disability [3.0% (*n* = 100)]. Among Hispanic adults, 8.1% reported a disability, most commonly mobility only disabilities [2.8% (*n* = 119)].

**Figure 1:**
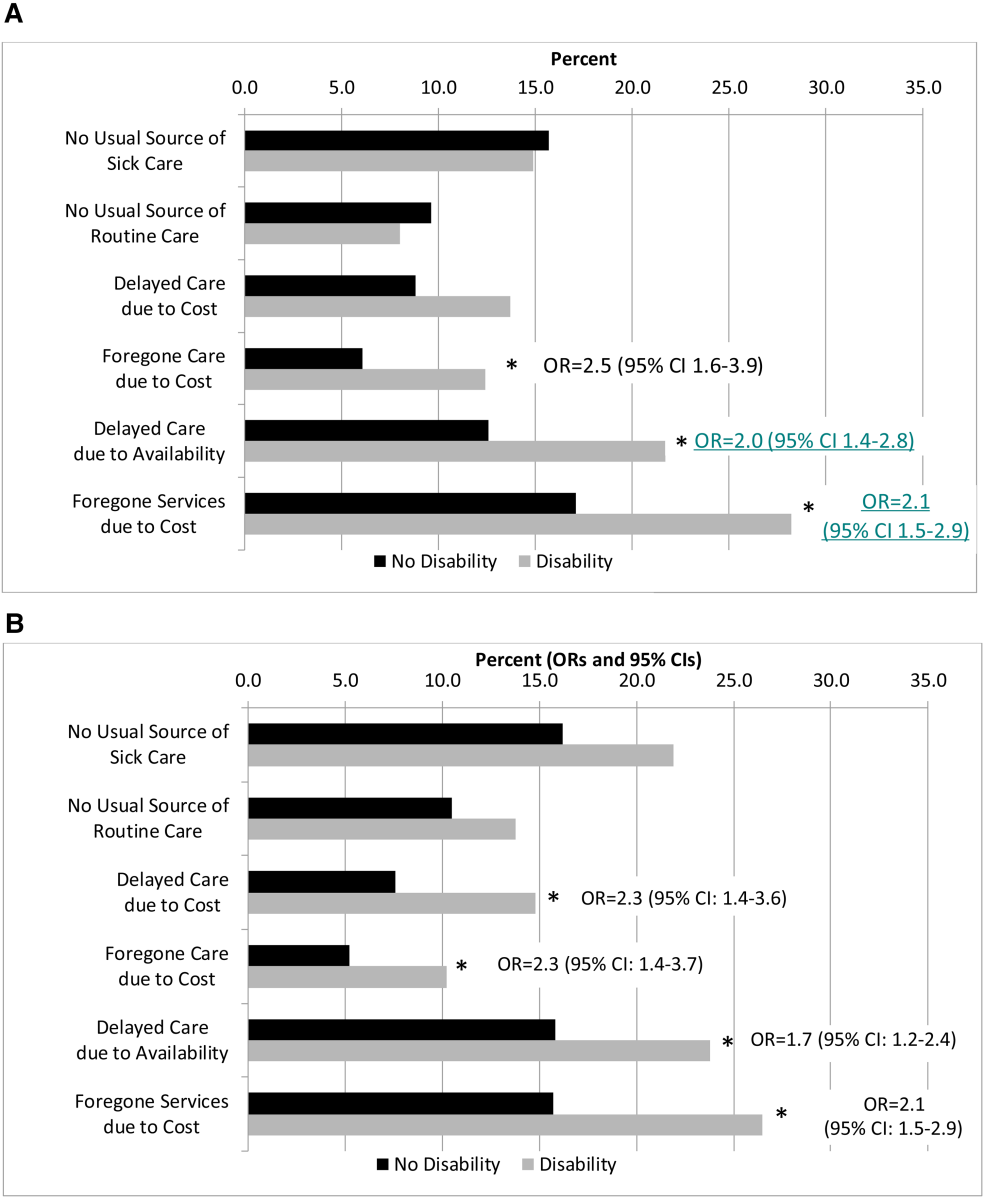
Predicted probabilities of access to healthcare by race/ethnicity and disability Status. (**A**) Black, non-Hispanic Adults. (**B**) Hispanic Adults. *p < 0.017, Indicates that the Odds ratios (OR) and 95% confidence intervals (CI) from logistic regression controlling for age, sex, marital status, employment status, educational attainment, poverty ratio, chronic conditions, and health insurance type. The ORs represent the disability group in reference to the no disability group.

The predicted proportions of access to care outcomes by race/ethnicity and disability are shown in Figure 1. Within each racial/ethnic group, PwD were significantly more likely to delay or forego care than their peers without disabilities. Nearly 30% of PwD forewent services due to cost (Black, non-Hispanic: 28.2%; Hispanic: 26.5%) compared to persons without disabilities (Black, non-Hispanic: 17.1%; Hispanic: 15.7%, both *p* < 0.001). There were no significant differences in access to usual source of care by disability group for either racial/ethnic group or for delaying care due to cost among Black, non-Hispanic adults.

## Discussion

We found that Black and Hispanic adults with disabilities were significantly more likely to forego or delay care due to reasons such as cost as compared to Black and Hispanic adults without disabilities. This suggests that BIPOC with disabilities experience greater disparities in access to healthcare than those without disabilities. This may reflect the systemic economic barriers experience by both BIPOC and disability communities, as well as those who experience intersectionality (10, 21). Policies and programs are needed to reduce costs whenever possible to ensure that BIPOC with disabilities (and BIPOC and PwD more generally) receive timely access to appropriate healthcare.

We found that across both Black and Hispanic populations, there was no difference in reporting a usual source of care by disability status. This is similar to the findings of a study using Medicare Expenditure Panel Survey data from 2002 to 2010 (22). This study found that Hispanics with basic activity limitations were the only group to be more likely to report lacking a usual source of care, as compared to other disability groups who are Hispanic.

Racism and ableism are often thought of as parallel systems of oppression that work separately, yet this notion ignores experiences at the intersection of BIPOC and PwD (8, 9). Our findings bring together separate bodies of scholarship that show that PwD and racial/ethnic minority populations experience disparities compared to their non-disabled and non-Hispanic white peers. Moreover, our findings highlight the need to account for the intersection of these identities in future analyses to appropriately tailor programs and policies. The health care disparities experienced by racial and ethnic minorities living with disabilities is likely influenced by systemic racism and systemic ableism factors.

There are limitations to our findings. We focused our analysis on Black, non-Hispanic adults and Hispanic adults with and without disabilities. Additional research should be conducted to investigate Asian and Indigenous populations. Additionally, due to sample size, we collapsed disability types. Differences may exist based on type of disability. Another limitation is the fact the findings are descriptive and do not consider longitudinal data or related contextual factors. Health disparities research is often examined through risk and not indicative of a strength-based approach. Finally, the term BIPOC does not account for or reflect the breadth of diversity among BIPOC individuals. More research should be done to acknowledge and examine the diversity across and within BIPOC PwD.

Future research should examine the intersection of social stressors such as racism and ableism and how they uniquely impact the health outcomes of BIPOC individuals. The disparity at the intersection of BIPOC and disability suggests disability access issues faced by BIPOC with disabilities cannot be solved without systemic reform of the healthcare delivery system. With respect to systemic challenges faced by BIPOC PwD and in line with other initiatives and recommendations to increase health equity for other marginalized groups, it is likely that systemic changes such as reducing costs associated with quality health care, increasing accessibility to health care, and building provider competency in the unique needs of racial and ethnic minority PwD may meaningfully improve health outcomes for this community. However, further qualitative examination of health needs from the perspective of members of this community is needed.

Meeting the health needs of BIPOC with disabilities is a matter of quality as much as it is equity. BIPOC with disabilities are likely to experience additional barriers such as racism, compounded by what is often an urgent or chronic need to maintain frequent consistent use of the healthcare system. Understanding health outcomes experienced by those who are members of both groups is critical to developing successful interventions aimed at improving health outcomes for this marginalized group.

## Data Availability

The datasets presented in this study can be found in online repositories. The names of the repository/repositories and accession number(s) can be found below: https://www.cdc.gov/nchs/nhis/nhis_2018_data_release.htm.
